# Tinea Incognito: Challenges in Diagnosis and Management

**DOI:** 10.3390/jcm13113267

**Published:** 2024-05-31

**Authors:** Aikaterini Zacharopoulou, Aikaterini Tsiogka, Antonios Tsimpidakis, Androniki Lamia, Dimitra Koumaki, Stamatios Gregoriou

**Affiliations:** 1 Department of Dermatology and Venereology, National and Kapodistrian University of Athens, Andreas Sygros Hospital, 16121 Athens, Greece; a.tsiogka@yahoo.com (A.T.); tsimpidakis.antonis@gmail.com (A.T.); androniki.lamia@gmail.com (A.L.); stamgreg@yahoo.gr (S.G.); 2 Dermatology Department, University Hospital of Heraklion, 71110 Heraklion, Greece; dkoumaki@yahoo.gr

**Keywords:** tinea incognito, fungal infection, dermatophyte infection, corticosteroid, tacrolimus, pimecrolimus

## Abstract

Tinea incognito is a dermatophyte infection with atypical features, due to the use of topical or systemic steroids or other immunosuppressive medications. Delayed diagnosis, spread of the infection to critical body surfaces, resistance to antifungal drugs, and increased costs due to prolonged hospitalization and multiple treatment regimens often complicate tinea incognito. It can affect individuals of all ages and genders, but it is more common in children. Atypical clinical appearance often necessitates differentiation from other diseases such as eczema, seborrheic dermatitis, lupus erythematosus, psoriasis, or other non-fungal skin conditions. The treatment of tinea incognito usually involves discontinuation of topical steroids or other immunosuppressive medications. Preventive measures and management of the underlying fungal infection are necessary and can be achieved with antifungal drugs. Patients should wear loose cotton clothes, use boiling water for laundry, and iron their clothing before wearing them. Additionally, they should avoid sharing bed linens, towels, clothes, and shoes. This review aims to raise awareness of tinea incognito among health practitioners, provide tips for detecting the disorder, include it in the differentials, and evaluate the available diagnostic procedures.

## 1. Introduction

Tinea incognito (TI) is a dermatophyte infection with atypical features due to treatment with a topical immunosuppressive agent or systemic steroids. The primary cause of TI is the use of a topical corticosteroid, which is prescribed by a medical professional for a pre-existing disorder or suggested for the treatment of a missed mycoses diagnosis [1,2]. Topical calcineurin inhibitors like tacrolimus ointment and pimecrolimus cream have also been known to cause TI due to their immunomodulating properties in the area of application. This type of fungal infection can slowly extend and mimic other cutaneous disorders, leading to a misdiagnosis and delayed or unsuccessful treatment. TI is also known as steroid-modified tinea, which has become an increasingly concerning pandemic. The term “incognito” refers to the masking of the clinical signs of a fungal infection [3].

Tinea incognito (TI) was first reported in 1968 by two UK dermatologists, Dr. Adrian Ive and Dr. Ronnie Marks. They observed a group of patients with unusual skin lesions, resembling seborrheic dermatitis, eczema, lichen ruber planus, scleroderma, folliculitis, rosacea, and psoriasis, that responded to usual treatments. One-third of the cases presented with typical ringworm characteristics. Upon further examination, they diagnosed an underlying fungal infection that was masked by the use of topical steroids or other immunosuppressive medications, justifying the term “tinea incognita”. Subsequent publications also employed the term “tinea incognita” [1]. Clinical variations in dermatophytosis can be attributed to the fungi’s invasive capacity, infection location, and humidity. The term “tinea atypica” is suggested for dermatophytosis without typical clinical features [4].

## 2. Epidemiology

The epidemiology of TI, which represents approximately 40% of dermatophytoses, is not well-established due to underreporting and misdiagnosis, despite being common and having a global distribution [1,5,6,7]. TI was first described in 1968 and there have been more cases reported in recent years [1,8]. It is more prevalent in tropical and subtropical regions due to favorable conditions for fungal growth, such as high humidity and temperature [9,10]. TI is a condition that can affect individuals of all ages and genders, ranging from 2 to 81 years old. However, it is more commonly found in children, as evidenced by a retrospective review of cases [11]. Another study of 818 cases of TI diagnosed in children at a referral hospital between 1977 and 2006 also supports the higher pediatric incidence. Additionally, young adults are also commonly susceptible to dermatophytosis [12,13]. Based on previous reports, it was found that males had a higher infection rate of dermatophytic fungi [14]. Immunocompromised patients and those with diabetes mellitus (DM) are at a higher risk of developing TI, especially tinea pedis and onychomycosis [10,14,15,16].

As topical corticosteroids are used to treat an increasing number of dermatologic diseases, the prevalence of TI has also risen [6,9,17]. Topical calcineurin inhibitors may be less of a risk factor for TI due to their limited use caused by high cost [9]. Tinea infections are caused by a type of fungi that thrive in warm and moist environments. Various factors such as sweating, abrasion, and maceration can contribute to the development of this disorder. Certain populations, such as people living in crowded or unsanitary conditions or those who participate in activities involving prolonged contact with water, like swimming or water sports, may have a higher prevalence of tinea infections [18,19,20,21].

*T. rubrum* is the most commonly isolated dermatophyte in ΤΙ, followed by *T. mentagrophytes*, *E. floccosum*, *M. canis*, *M. gypseum*, *T. violaceum*, *T. tonsuran*, *T. verrucosum*, *T. schonleini*, and *T. erinacei* [21,22,23,24,25,26,27,28,29,30,31]. However, *T. rubrum* and *T. interdigitale* are the most common fungi for dermatophytosis in several regions [4,32]. Consequently, the question of whether the epidemiology of TI differs from tinea epidemiology in general is difficult answer. The prevalent strain in India has been reported to be *T. interdigitale* [33]. A few studies found *T. mentagrophytes* (44.4%) and *T. verrucosum* (33%) to also be frequently isolated species [31,34]. *M. canis* has also been reported to be common in one study [4].

The exact prevalence of TI among immunocompromised patients, particularly those with HIV, is not currently known. It may even be underestimated, especially in developed countries where antiretroviral treatment is initiated early and antifungal medication is prescribed more frequently for other mycotic disorders [35]. *M. gypseum* is a type of fungus that has been found in HIV patients, particularly in areas with limited resources [36,37,38,39]. Exposure to humid soil and dust may also increase the likelihood of developing an *M. gypseum* infection [40].

## 3. Pathogenesis

**Figure 1:**
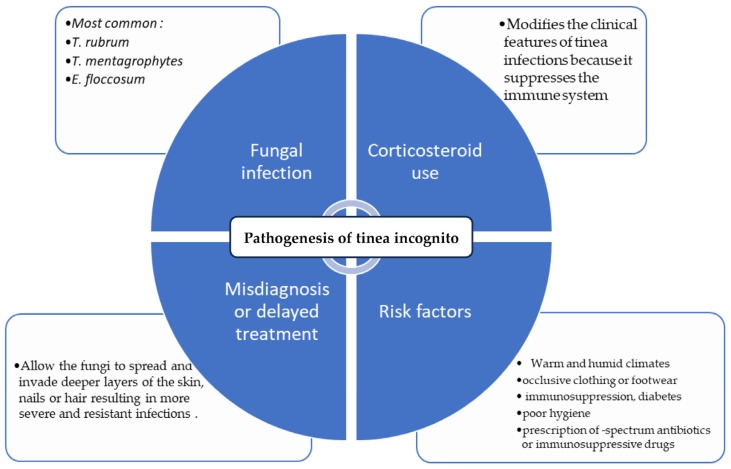
The pathogenesis of tinea incognita [1,5,9,10,15,23,25,30,43,44,45,46].

The development of TI results from the interaction of fungal infection, corticosteroid use, misdiagnosis and/or inappropriate treatment, as well as individual and environmental factors that affect the sensitivity and severity of the infection (Figure 1). The risk factors that increase the likelihood of developing dermatophytosis include a weakened immune system and damaged skin [41,42]. The diminished nutrient availability with concomitant reduced oxygenation and antimicrobial peptide dysfunction (AMP) at the infection site might also contribute to the risk factors for the initial infection.

**Figure 2:**
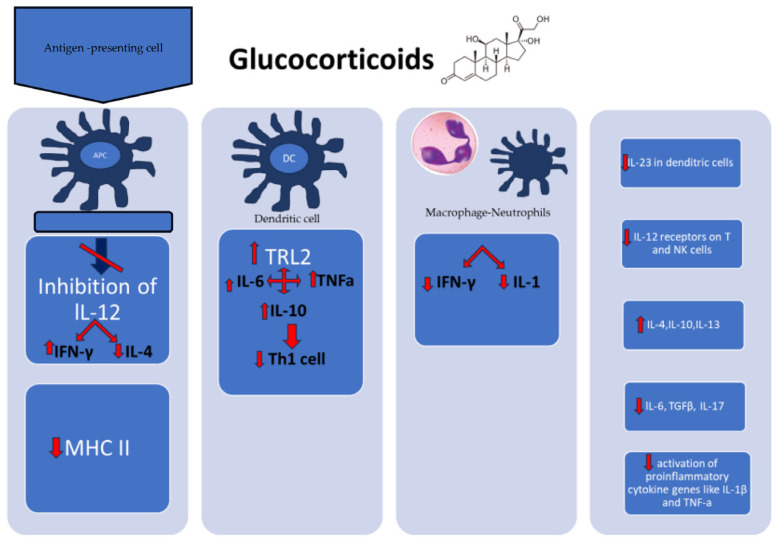
The effect of topical or systemic glucocorticoids on pathogenesis of tinea [49]. Corticosteroids act on APCs, suppressing the inhibition of lL-12 and decreasing MHC II expression. Furthermore, exposure of dendritic cell to corticosteroids induces the expression and secretion of IL-6, IL-10, and TNFa through TLR2 activation, which leads to inhibition of Th1 cell activation. Corticosteroid application shifts the immune response towards a Th2 response. This is mediated through downregulation of IL-12 receptors on T and NK cells and via inhibition of IL-12 production. Th17 differentiation and function may be affected by corticosteroids. Corticosteroids decrease the expression of IL-23 in denditric cells and lL-6, TGFβ, IL-17. Additionally, corticosteroids upregulate production of IL-4, IL-10, IL-13 and directly or indirectly suppress the activation of proinflammatory cytokine genes like IL-1β and TNF-a. Finally, corticosteroids reduce macrophage and neutrophil recruitment and IL-1 and IFN-γ release by macrophages [46]. APC—artigen presenting cell; IFN-γ—interferon gamma; IL—interleukin; MHC—major histocompatibility complex; NK cell—natural killer cell; TGFβ—transforming growth factor beta; Th—T helper; TLR—toll-like receptor; TNF—tumor necrosis factor.

TI and uncomplicated tinea infections are typically caused by the same pathogens, such as dermatophytes *T. rubrum*, *T. mentagrophytes*, and *E. floccosum* [47]. The pathogenesis of TI is distinct from traditional tinea infections because it is altered by the use of topical steroid creams, systemic corticosteroids, or other immunosuppressive medications [48]. These medications, particularly when used frequently or in high doses, can suppress normal immune responses, and reduce the inflammation limiting fungal infections. This may contribute to the growth and spread of the infection (Figure 2) [4,5,14]. Topical steroids may also mask the symptoms of a fungal infection, such as redness, itching, and scaling, making the infection appear less severe [9]. If left undiagnosed or misdiagnosed as another skin disorder, a fungal infection may not be treated in a timely manner. This can result in a more severe and recurrent infection that is harder to treat. In such cases, oral antifungal medications may be required for a more aggressive treatment approach [25].

## 4. Clinical Features

**Figure 3:**
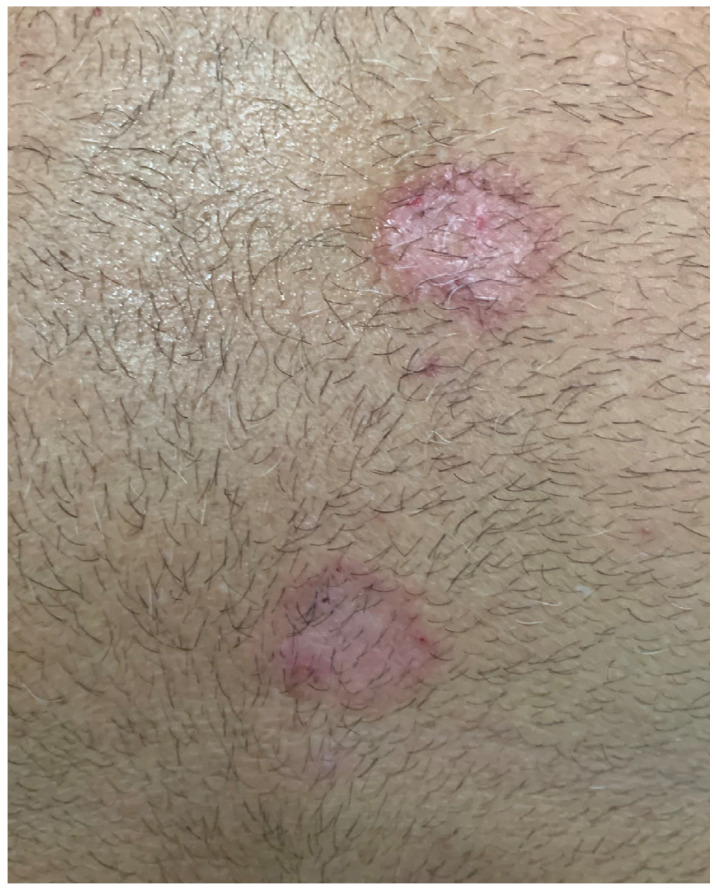
Tinea incognito treated as nummular eczema with topical steroids.

A typical scenario begins with a misdiagnosis of TI as eczema (Figure 3), leading to the use of topical steroids that reduce inflammation and make the condition less irritable [5,9,46]. The more topical steroids are applied, the more extensive and unrecognizable the fungal infection becomes. Furthermore, long-term use of topical steroids can cause skin atrophy, which complicates the diagnosis of TI [9]. Thinning of the skin caused by atrophy may resemble the chronic phase of other skin conditions that are treated with steroids for a prolonged period, such as eczema, lupus erythematosus, psoriasis, or other non-fungal skin conditions [23,30,45]. As topical steroid application continues, it becomes increasingly challenging to differentiate between these conditions and tinea incognito (TI). Delayed treatment may allow the fungi to spread and invade deeper layers of the skin, nails, or hair, resulting in more severe and resistant infections.

A variation of the typical scenario involves the use of a combination of topical steroid and antifungal cream. Even though such a combination compounds is never an adequate option in the treatment of dermatophyte infections, over-the-counter purchase by patients or misguided prescription by physicians occurs in real-world practice. In such cases and particularly in areas of occlusion, such as the buttocks, groin, and armpit, the impaired immunity caused by the topical steroid might be sufficient to cause TI, despite the application of the topical antifungal.

**Figure 4:**
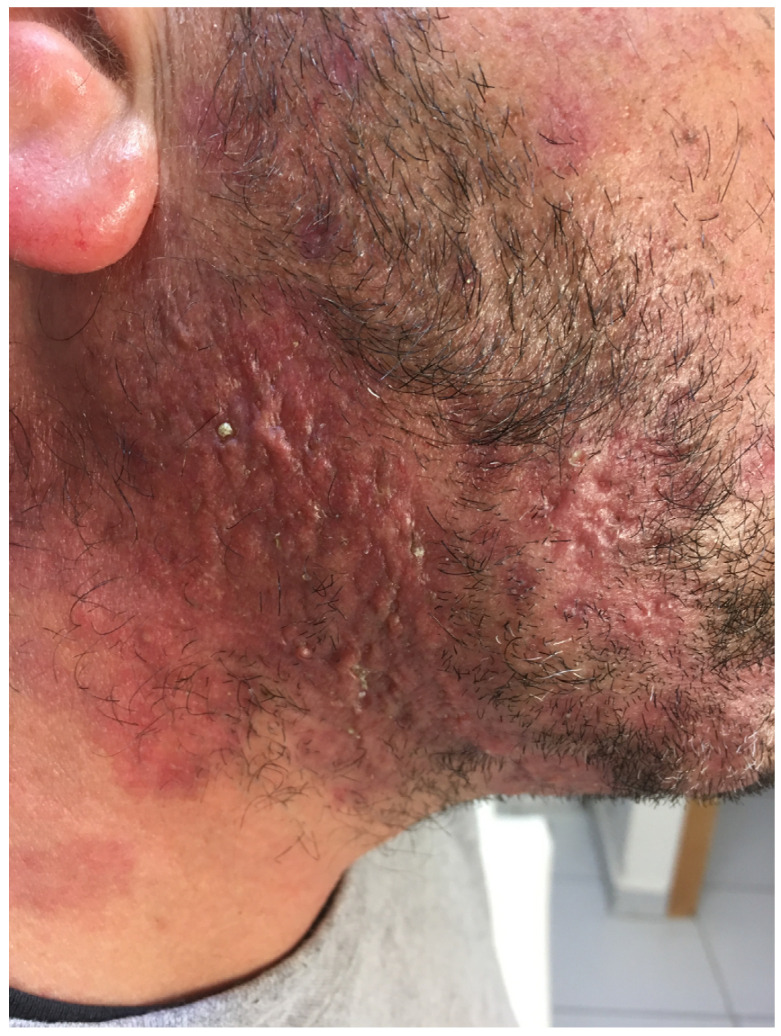
Tinea incognito treated with topical steroids for three months resulting in scar formation.

**Figure 5:**
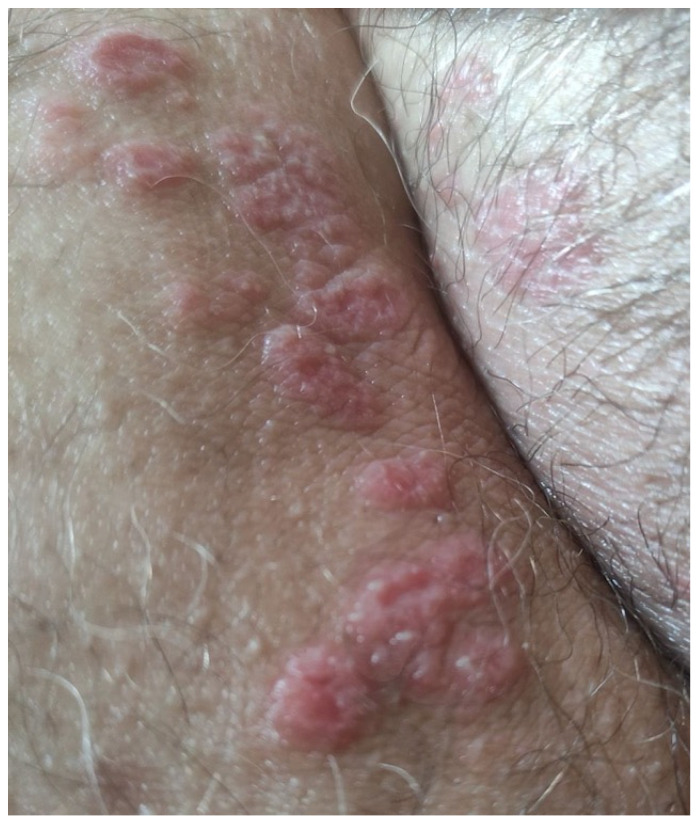
Dermatophyte infection treated with topical combination cream of topical steroid plus topical fusidic acid.

TI is a type of fungal skin infection that can present with various and non-specific clinical features, such as a sudden onset of pruritus in a previously unaffected area. The symptoms depend on the type of the underlying fungus and the location of the infected body site. Lesions might appear larger, diffuse, poorly defined, less red and scaly, and more pinkish or flesh-colored [21,23,31]. Table 1 provides a summary of various clinical conditions that need to be distinguished from TI (Figure 4 and Figure 5).

A typical dermatophytosis usually begins as a red circular plaque with a slightly raised scaly border. As the plaque expands, it takes a ring shape with scales at the periphery [8,15]. In time, multiple itchy lesions can develop and coalesce into patches. These patches may exhibit a poorly defined border, florid growth, and loss of characteristic features due to inflammation suppression [26].

TI often results in a red, scaly rash that can be itchy, uncomfortable, or painful. The rash may appear in patches or spread over a larger area on any part of the body, but it is usually seen in areas where skin folds, such as the groin, the axillae, and the inframammary region. Unlike an untreated fungal infection, TI’s margin is less elevated, less scaly, and more commonly pustular, and the typical “ring-like” shape may be absent. Next to the initial plaque, secondary lesions may emerge. Sometimes, a secondary bacterial infection may occur, showing concurrent pustules and impetigo. In some cases, the rash may also be accompanied by blisters or pustules that are filled with fluid and can break, open, and ooze [8,15]. The affected skin may appear lighter or darker than the surrounding skin due to erythematous or hyperpigmented lesions [25]. In chronic infections, the skin may thicken or develop a leathery texture. It can also cause hair loss due to persistent dermatophytic infection and possible invasion of deeper tissues and hair follicles

A useful clinical tip to differentiate between bacterial infections and TI complicated by a secondary bacterial infection is that in the latter case, hairs can be pulled out of the follicle without the patient feeling any pain.

In several tinea infection cases, the borders of the scaly plaques might not be well established, and new satellite lesions might appear beyond the blurred border of the initial lesion, due to suppression of the inflammatory reaction at the periphery. More often, especially in the initial stages of the disease, the central remission of the lesion and the presence of a faintly marked ellipsoidal or semi-annular border peripherally are observed. This should raise the suspicion of underlying dermatophytes.

Tinea infections might also develop inflammatory nodules [26] Concentric circles sometimes appear during fungal infections, which can indicate repeated episodes of fungal expansion. Alongside clinical variations caused by topical agents, systemic immunosuppression can make the condition worse, leading to deep subcutaneous abscesses. This can cause the formation of a secondary granuloma, which is known as “Majocchi’s granuloma”. Majocchi granuloma is a skin infection caused by fungi that typically affect hair follicles. The most common causative agents are Trichophyton rubrum and Microsporum canis. The infection occurs when the fungi spread into the surrounding tissues after an injury to a hair follicle. This condition is rare. It results in the formation of nodular granulomatous perifolliculitis, which is characterized by an intense inflammatory response. Clinically, lesions appear as granulomata, cellulitis, or plaques. They are often seen on the anterior aspect of the legs, but inguinal, scrotal, vulval, and facial involvement has also been reported. In some cases, patients may have an underlying immunodeficiency state, particularly due to corticosteroid therapy, which mainly affects delayed hypersensitivity reactions. Disseminated skin lesions may rarely occur in immunosuppressed individuals [50,51].

In some cases, the fungal infection can persist for months or years, become systemic, and even lead to onychomycosis. In cases of extensive dermatophyte infection, the patient’s sexual partners should also be checked for the infection. Although extremely rare, an id-reaction may occur in TI.

## 5. Diagnosis

Diagnosing tinea incognito (TI) can be quite challenging due to the atypical appearance of the lesions. Physical examination of the affected skin is usually necessary to detect signs of a fungal infection, such as scaling and redness.

Dermoscopy, a non-invasive technique, might assist in the diagnosis when suspecting TI. Dermoscopic features observed in tinea corporis and tinea capitis might be prevalent in TI, after topical treatment is discontinued, and help in diagnosis, although literature data are lacking. The most commonly observed dermoscopic findings in tinea corporis cases include dotted vessels, superficial white scales with peripheral distribution, and the presence of a ‘moth-eaten’ scale with an outward-peeling direction, which appears to be the most specific feature [52]. The most common trichoscopic findings in tinea capitis include comma hairs, corkscrew hairs, zigzag hairs, and Morse code hairs. In addition, black dots and broken hairs are frequently seen. Successful treatment of tinea capitis is characterized by a reduction in short broken hairs, corkscrew and zigzag hairs, and later, comma hairs and black dots [53].

**Figure 6:**
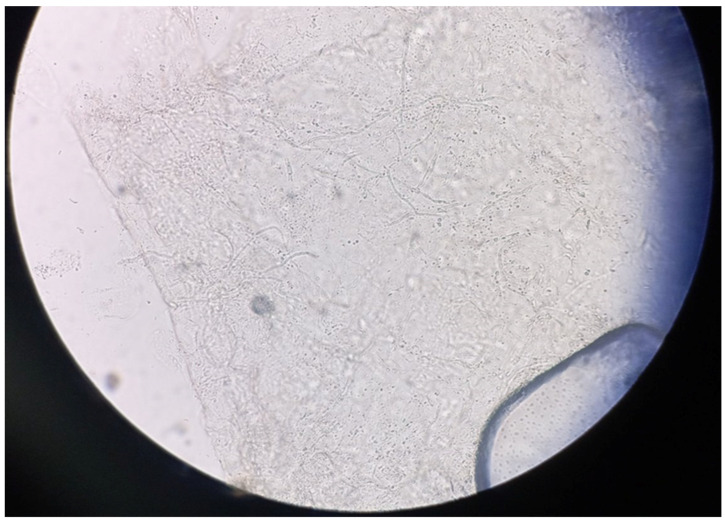
KOH preparation of fungal elements.

Confirmation of TI diagnosis requires mycological examination after discontinuing topical steroids. To prevent bacterial growth, skin scales should be collected from the periphery of the plaque and kept dry [54,55,56]. Direct microscopic examination is a quick, simple, and affordable method for screening superficial mycosis. A 10–40% potassium hydroxide solution (KOH-10% for skin scrapings and 40% for nail clippings) is applied. Positive scrapings are indicated by the presence of hyphal filaments, with or without arthroconidiospores. Hyphae can be seen between epithelial cells [53]. The accuracy of the diagnosis depends on the quality of the sample and the skill of the physician [54]. Adding either 36% dimethyl sulfoxide (DMSO) or Chicago Sky Blue stain to the KOH solution will stain the fungal hyphae blue against a pink background, providing better visualization of the fungal elements [57]. Fluorescent staining using diaminostilbene is the most sensitive microscopic method for specimen visualization [58]. Fluorescent microscopy combined with Calcofluor or Blankophor improves identification speed, ease, and safety [59]. During fluorescent microscopy, fungal filaments and spores appear as blue-white when exposed to ultraviolet light. Chorazol black E staining increases specificity to about 98% [60] (Figure 6).

Fungal culture is the most reliable method to diagnose TI, but it may not always be accessible [55]. Utilization is important, especially in stubborn or severe cases [61]. To identify the fungus responsible for an infection, experts rely on the appearance of the colonies.

In cases where a person is suspected to have tinea, a skin or nail biopsy may be necessary to confirm the diagnosis. The biopsy will be examined under a microscope using stains such as periodic acid–Schiff (PAS) or Gomori–Grocott methenamine silver, which will highlight any fungal elements present. Although the biopsy appearance may vary, certain elements support the diagnosis of tinea. The histopathology of tinea incognito is similar to other fungal infections, with visible fungal hyphae between cornified cells in the stratum corneum (also known as the ‘sandwich sign’), compact orthokeratosis, and neutrophils in the stratum corneum [62,63].

Polymerase chain reaction (PCR) and nucleic acid sequence-based amplification are two of the more recent diagnostic tools [45]. Uniplex PCR can detect and identify fungi with overlapping characteristics of culture, sensitivity, and specificity [64]. Multiplex PCR can detect 21 dermatomycotic pathogens with DNA using agarose gel electrophoresis. Other techniques include gene-specific PCR, sequencing of rRNA gene, chitin synthase encoding gene, PCR fingerprinting, and DNA hybridization [64].

The matrix-assisted laser desorption ionization–time of flight mass spectrometry (MALDI-TOF-MS) is a diagnostic technique that detects proteolytic degradation products resulting from fungal activity [54]. Reflectance confocal microscopy is another method used for diagnosing tinea. It offers in vivo imaging of the epidermis and superficial dermis at the cellular level to detect fungal infections [65,66].

## 6. Treatment

The treatment of TI typically involves stopping the use of topical steroids or other immunosuppressive medications. Preventive measures and management of the underlying fungal infection are necessary and can be achieved with antifungal medications. The literature suggests that discontinuing immunosuppressive and classic antifungal regimens is sufficient to treat TI. Patients should wear loose cotton clothes, use boiling water for laundry, and iron their clothing before wearing them. Additionally, they should avoid sharing bed linens, towels, clothes, and shoes. Obese patients or those with hyperhidrosis should be encouraged to lose weight and apply topical aluminum salts or anticholinergics to reduce sweating.

The ideal pharmacological treatment should be highly effective and provide long-term prophylaxis against relapses. It should also have potent anti-inflammatory action, minimal adverse events, low cost, and be safe to use in pregnancy and lactation, as well as renal and hepatic failure. Treatment modalities include oral or topical antifungals or a combination of both, depending on the severity and extent of infection, as well as the type of the culprit fungi [3]. Combinations of various antifungal groups should be used to prevent resistance from emerging.

Topical antifungal medications are typically the first line of treatment for mild cases of tinea infections and for localized and primary dermatophytic infections. Antifungal creams, gels, or lotions, which contain agents like terbinafine, clotrimazole, miconazole, or econazole, can be used to treat fungal infections. The medication is applied to the affected area(s) twice daily for several weeks until the infection disappears. Treatment typically lasts for several weeks. Additionally, bland antipruritic lotions can also be applied [44].

In cases where the infection is severe or widespread or involves hair-bearing areas, oral antifungal medications may be prescribed. Terbinafine, itraconazole, and fluconazole are medications that have been shown to be more effective than griseofulvin. Treatment duration varies depending on infection severity and patient response, but usually lasts for several weeks to several months (Table 2) [44].

Systemic antifungal agents can have adverse effects. Itraconazole may lead to elevated liver enzymes and gastrointestinal issues [29]. Terbinafine has been linked to a condition called neutropenia or agranulocytosis, which is a reduction in the number of white blood cells [28]. Laboratory evaluation should be considered before starting treatment, and monitoring should be performed regularly. It is important to review the patient’s medical history because systemic antifungals can interact with other medications prescribed for concomitant disorders.

The emergence of resistance to antifungal drugs is becoming a problem [67]. Information on dermatophyte resistance is limited compared to systemic infections [67,68,69,70]. There is a need for novel therapeutic agents that can target the mechanisms by which fungi limit the immune response and promote the re-establishment of cell-mediated immunity [49].

## 7. Recurrence

Patients may purchase combination formulations (steroids with antifungal and antibacterial agents) or over-the-counter medications to prevent recurrence [9,21]. The literature suggests that most TI patients self-treat or rely on advice from friends and family for treatment [21,31]. Using these modalities can actually increase the risk of a recurrence instead of providing preventative measures.

Physicians should emphasize the importance of completing the prescribed treatment in order to completely eradicate the infection, even if the symptoms improve earlier. Follow-up visits are important to ensure that the treatment was effective and to prevent the possibility of relapse. It has been reported that patients with impaired epidermal barrier functionality, such as those with atopic dermatitis, those with immunosuppression due to malignancies or because of immunosuppressive therapy, are at higher risk of recurrent infections [71,72,73].

## 8. Financial Considerations

It has been observed that TI leads to an increase in the number of consultations and laboratory investigations. It is possible that treatment for dermatophytosis may take longer and require more expensive antifungal medication. In India, a recent analysis of treatment costs showed that patients who use topical steroids spend 40% more on treatment than those who do not use steroids. This was true for both patients who had never used steroids before and those who had used them in the past [3]. The epidemiology of dermatophytosis in India indicates that the high temperatures and humidity prevalent in the region promote the acquisition and perpetuation of fungal infections. Therefore, these results may not be applicable to other regions with different climatic conditions. However, the additional costs associated with the treatment of these infections should raise concerns regarding the increased morbidity and economic burden in all geographical settings.

## 9. Conclusions

The clinical presentation of tinea incognito (TI) can be unusual and highly variable. The lesions may not appear as expected for a typical fungal infection, making it difficult to diagnose and treat. Furthermore, the use of topical steroids or other immunosuppressive medications may make the affected skin thinner and more fragile, increasing the risk of injury or secondary bacterial infections. Misdiagnosis of TI can result in delayed treatment with subsequent complications and increased economic costs due to hospitalization. Therefore, diagnosing TI requires a high index of suspicion. Additional tests such as skin scrapings, cultures, or other diagnostic tests may be necessary to confirm the presence of a fungal infection. Early and accurate diagnosis is crucial to prevent the spread of infection and ensure that appropriate antifungal treatment is administered.

## Figures and Tables

**Table 1:** Differential diagnosis of tinea incognito [21,31].

Granuloma Annulare	Rosacea
**Discoid Lupus Erythematosus**	Morphea
**Pityriasis rosea**	Lichen Planus
**Perioral Dermatitis**	Folliculitis
**Seborrheic Dermatitis**	Impetigo
**Erythema Annulare Centrifugum**	Scaly Achromatic Plaques
**Irritant Contact Dermatitis**	Scattered/Extensive Erythematous Plaques
**Nummular Eczema**	Erythematous Plaques of Concentric Circles
**Purpura**	Orificial Granulomatous Dermatitis
**Psoriasis Vulgaris**	Atypical Annular Lesions
**Pustular psoriasis**	Pustular, Inflammatory Lesions
**Pyoderma**	Ear-Face Erythema

**Table 2:** Treatment of tinea.

Agent/Class	Route	Preparations	Frequancy of Application/Dosage Forms		Duration of Treatment
			Adult Dose	Pediatric Dosing per kg Body Weight Dose	
**Tinea Corporis/Curis**					
**Imidazoles**					
**Clotrimazole 1%**	Topical	Cream, oint., solution, lotion, powder	Twice daily		4–6 weeks
**Econazole 1%**		Cream, spray	Once daily		2–4 weeks
**Miconazole 2%**		Cream, spray, gel, oint., solution, powder, tincture	Twice daily		4–6 weeks
**Oxiconazole 2%**		Cream, lotion	Once or twice/daily		At least 2 weeks
**Sertaconazole 2%**		Cream	Twice daily		4 weeks
**Luliconazole 1%**		Cream, lotion	Once daily		1–2 weeks
**Eberconazole 1%**		Cream	Twice daily		2–4 weeks
**Ketoconazole 2%**		Cream, shampoo, gel, foam	Once daily		2 weeks
**Allylamines**					
**Terbinafine**	Topical	Cream, gel, spray, solution (1%)	once or twice daily		2 weeks
	Oral	Tablets	250 mg/day or 125 mg/twice daily	<20 kg (>2 years old): 62.5 mg/once/daily 20–40 kg: 125 mg once daily >40 kg: 250 mg once daily	2–4 weeks
**Naftifine**	Topical	Cream (1%), gel (1–2%)	once daily (cr.), twice daily (gel)		2–4 weeks
**Butenafine 1%**	Topical	Cream	Once daily		2 weeks
**Morpholines**					
**Amorolphine 0** **.** **25%**	Topical	Cream	twice weekly		2–3 weeks
**Triazoles**					
**Itraconazole**	Oral	Caps/tablets	100 mg/day/2 weeks 200 mg/day/1 week	3–5 mg/kg/day/1 week	1–2 weeks
**Fluconazole**	Oral	Caps	150–200 mg/week 50–100 mg/day	6 mg/kg/week	2–6 weeks
**Heterocyclic benzofuran**					
**Griseofulvin**	Oral		500–1000 mg/day (microsize sus-pension) 300–375 mg/day (ultramicrosize suspension)	15–20 mg/kg/day (microsize sus-pension) 10–15 mg/kg/day (ultramicrosize suspension)	2–4 weeks
**Tinea pedis/Manuum**					
**Imidazoles**	Use the same dose as Tinea Corporis, except for topical ketoconazole 2%, which should be applied once daily for 6 weeks.				
**Allylamines**					
**Terbinafine**	Topical	Cream, gel, spray, solution (1%)	Once–twice/day		2–6 weeks
	Oral	Tablets	250 mg/once daily		2–4 weeks
**Naftifine 1%**	Same with Tinea Corporis				
**Butenafine 1%**	Same with Tinea Corporis				
**Morpholines**					
**Amorolphine 0.25%**	Topical	Cream	2 weekly		Up to 6 weeks
**Triazoles**					
**Itraconazole**	Oral	Caps/tablets	100–200 mg/day	3–5 mg/kg/day	2–4 weeks
**Fluconazole**	Oral	Caps	150 mg/week	6 mg/kg/week	4 week
**Efinaconazole 10%**	Topical	Solution	Once day		48 weeks
**Heterocyclic benzofuran**					
**Griseofulvin**	Oral		750–1000 mg/day (microsize sus-pension) 660–750 mg/day (ultramicrosize suspension)	15–20 mg/kg/day (microsize sus-pension) 10–15 mg/kg/day (ultramicrosize suspension)	4–8 weeks
**Others**					
**Ciclopirox 0.77%**	Topical	Cream/gel	Twice daily		4 weeks
**Tinea Capitis**					
**Allylamines**					
**Terbinafine**	Oral	Tablets	250 mg once daily	<25 kg (>4 years old): 125 mg/once/daily 25–35 kg: 187.5 mg once daily >35 kg: 250 mg once daily	3–4 weeks
**Triazoles**					
**Fluconazole**	Oral	Tablets	Daily dosing: 6 mg/kg/day Weekly dosing: 6 mg/kg/week	6 mg/kg/day	3–6 week for daily dosing 8–12 weeks for weekly dosing
**Itraconazole**	Oral	Solution/capsules	Caps: 5 mg/kg/day Solution: 3 mg/kg/day Pulse therapy with caps 5 mg/kg/day for one week each month/2–3 months Pulse therapy with oral solution: 3 mg/kg/day for one week each month/2–3 months	5 mg/kg/day	4–8 week
**Heterocyclic benzofuran**					
**Griseofulvin**			20–25 mg/kg/day (microsize sus-pension-maximum 1 g/day) 10–15 mg/kg/day (ultramicrosize suspension-maximum dose: 750 mg/day)	20–25 mg/kg/day (microsize sus-pension) 10–15 mg/kg/day (ultramicrosize suspension)	6–12 weeks (continue for 2 weeks after symptoms and sings have resolved)
**Laboratory monitoring**					
**Griseofulvin**	No baseline testing. If required for longer than 8 weeks: ALT, AST, BILIRUBIN, CREATINIE measurements and CBC every 8 weeks				
**Terbinafine**	Baseline ALT and AST measurement. CBC at 6 weeks forcourses lasting longer than 6 weeks				
**Itraconazole**	Baseline ALT and AST measurement				
**Fluconazole**	Baseline ALT, AST, and CREATININE measurement and CBC				

## Data Availability

The data that support the findings of this study are openly available in MEDLINE electronic database.
